# Patient perspective of tardive dyskinesia: results from a social media listening study

**DOI:** 10.1186/s12888-021-03074-9

**Published:** 2021-02-15

**Authors:** Mallory Farrar, Leslie Lundt, Ericha Franey, Chuck Yonan

**Affiliations:** Neurocrine Biosciences, Inc., 12780 El Camino Real, San Diego, CA 92130 USA

**Keywords:** Social media listening, Tardive dyskinesia, Patient perspective, Disease burden, Antipsychotics

## Abstract

**Background:**

Tardive dyskinesia (TD) is a persistent and potentially disabling movement disorder associated with prolonged exposure to dopamine receptor blocking agents such as antipsychotics. With the expanding use of antipsychotics, research is needed to better understand patient perspectives of TD, which clinical assessments may fail to capture. Social media listening (SML), which is recognized by the US FDA as a method that can advance ongoing efforts for more patient-focused drug development, has been used to understand patient experiences in other disease states. This is the first study to use SML analysis of unsolicited patient and caregiver insights to help clinicians understand how patients describe their symptoms, the emotional distress associated with TD, and the impact on caregivers.

**Methods:**

In this pilot study, a comprehensive search was performed for publicly available, English-language, online content posted between March 2017 and November 2019 on social media platforms, blogs, and forums. An analytics platform (NetBase™) identified posts containing patient or caregiver experiences of assumed TD using predefined search terms. All posts were manually curated and reviewed to ensure quality and validity of the post and to further classify key symptoms, sentiments, and themes.

**Results:**

A total of 261 posts from patients/caregivers (“patient insights”) were identified using predefined search terms; 107 posts were used for these analyses. Posts were primarily from forums (47%) and Twitter (33%). Analysis of the most common sentiment-related terms (e.g. “feel” [*n* = 31], “worse” [*n* = 17], “symptom” [*n* = 14], “better” [*n* = 12]) indicated that 64% were negative, 33% were neutral, and 3% were positive. Theme analysis revealed that patients often felt angry about having TD from a medication used to treat a different condition. In addition, patients felt insecure, including feeling unaccepted by society and fear of being judged by others.

**Conclusion:**

Although this study was limited by inherent methodological constraints (e.g., small sample size, reliance on patient self-report), the perspectives generated from analyzing social media may help convey the unmet needs of patients with TD. This analysis indicated that movement-related symptoms are the most common patient concern, resulting in strong feelings of anger and insecurity.

**Supplementary Information:**

The online version contains supplementary material available at 10.1186/s12888-021-03074-9.

## Background

Tardive dyskinesia (TD) is a persistent and potentially disabling movement disorder associated with prolonged exposure to dopamine receptor blocking agents such as antipsychotics [1]. TD is characterized by abnormal and involuntary movements in one or more body regions, including the head, face, neck, trunk, hips, limbs, and/or extremities. When second-generation antipsychotics (SGAs) were developed, it was hoped that these newer drugs would reduce the risk for TD. A meta-analysis of antipsychotic studies published from 2000 to 2015 estimated that the global mean prevalence of TD was lower with SGAs (20.7% vs 30.0% for first-generation antipsychotics [FGAs]), but the authors also noted that the introduction of SGAs did not mitigate the risk for TD as previously thought and/or expected [2]. With antipsychotics being approved for additional non-psychotic psychiatric conditions, along with increased off-label use, the risk of TD continues to be a clinical concern [3].

TD has been shown to negatively affect daily functioning and health-related quality of life (QoL) [4, 5]. The burden of TD may be even more pronounced in individuals who are highly functional (e.g., able to work) and aware of their movements [6]. Effective treatments are needed for all TD patients, but they may be particularly important to individuals who are more aware of their TD symptoms and/or bothered by those symptoms. In its updated clinical practice guidelines for schizophrenia, the American Psychiatric Association (APA) recommends that reversible vesicular monoamine transporter 2 (VMAT2) inhibitors (e.g., valbenazine or deutetrabenazine) be used to treat moderate or severe TD [7]. For milder forms of TD, the APA states that VMAT2 inhibitors can be considered based on factors such as patient preference, TD-related impairment, and impact on psychosocial functioning.

For VMAT2 inhibitors currently approved by the FDA for TD, registration trials included clinician-rated assessments such as the Abnormal Involuntary Movement Scale and patient-reported outcomes (PROs) such as the Patient Global Impression of Change (PGIC) [8–11]. However, these trials and scales were not designed to capture the emotional burden of TD on individual patients. Social media listening (SML) is a method that allows for the analysis of unsolicited patient insights that are not normally captured in clinical trials. Moreover, SML analyses can represent the experiences of individuals who may be unlikely to participate in a clinical trial or patient preference study.

SML has been used to understand patient experiences in other therapeutic areas [12, 13], and final 2020 guidelines from the FDA lists social media as an appropriate method for gathering patient input [14]. In addition, the International Society for Pharmacoeconomics and Outcomes Research notes that collecting data from social media sources can help bring attention to the issues that are most relevant to patients and their families [15]. Given the need for more patient-oriented research in TD, this study was conducted to analyze the emotional content of online posts from patients with presumed TD.

## Methods

### Study design and data sources

A comprehensive search of social media content (e.g., Twitter, blogs, online forums) was conducted using an analytics program that uses artificial intelligence and natural language processing to “understand” the sentiment of online posts and group those sentiments into themes (NetBase™; Santa Clara, California; www.netbase.com). English-language content from the United States posted online from 01-Mar-2017 to 07-Nov-2019 was searched using the following predefined terms: “tardive dyskinesia” or spelling variations (e.g. “tardarive diskensia) (Supplementary Table S1). Posts that were overtly political or included specific medications (brand or generic name) were excluded (Supplementary Table S1).

All posts were collected from publicly available sites that were not password-protected. Social media platforms that do not provide public access to posts (e.g., Facebook) were excluded from the search. To protect the privacy of “users” (i.e., individuals who posted social media content), all personal information was anonymized prior to any manual review of the content. Verbatim quotes from posts are not presented to further protect the privacy of users. An ethics committee for the study sponsor, Neurocrine Biosciences, Inc. (San Diego, California), approved the study design. As a retrospective analysis based on posts from publicly available social media sources, no approval from an Institutional Review Board was required or obtained. However, the study was conducted in accordance with International Society for Pharmacoeconomics and Outcomes Research guidelines for studies pertaining to health economics and outcomes research [15].

### Selection and characterization of posts

Search results were manually reviewed by MF, EF, and LL for relevance. A unique identification number was assigned to each post included in this analysis. Multiple posts from individual users were allowed. Based on available information, whether explicitly stated or inferred from content, posts were analyzed for gender (per reviewer judgement based on screen names/pictures when available) and type of user (patient or caregiver, per reviewer judgment based on content of posts). Sources for the posts were documented (e.g., Twitter). Web addresses of posts were provided when available; screenshots were provided when links were inaccessible.

### Sentiment and theme analyses

The NetBase™ analytics program was used for the following procedures: 1) develop a list of words/phrases within selected posts that described users’ experience with TD; 2) tabulate the number of times that these terms were mentioned; 3) identify the sentiment of the terms as “negative”, “positive”, or “neutral”; and 4) group the sentiments into larger themes. The analytics program was able to recognize linguistic complexities such as tone (e.g., sarcasm), cultural references, slang words, and emojis. The terms, sentiments, and themes generated by the analytics program were manually reviewed for quality and validity.

### Statistical analysis

Quantitative data (e.g., demographics, user type, social media source, tabulation of terms, sentiment analysis) were aggregated and analyzed descriptively.

## Results

### Selection and characterization of posts

**Fig. 1:**
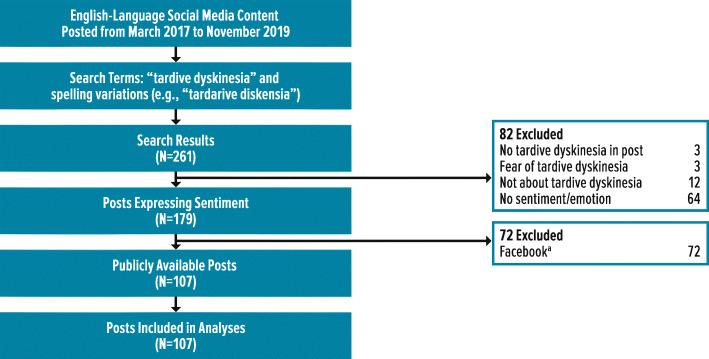
Selection of Posts. ^a^Social media platforms which did not provide public access to information/posts (e.g., Facebook) were excluded

A total of 261 posts were identified using the predefined search terms (Fig. 1). Of these, 154 were excluded for being non-relevant to TD or lacking sentiment and/or emotion (*n* = 82) or because the social media platform does not allow public access to posts (e.g., Facebook) (*n* = 72). The remaining 107 posts were included for analyses, including for sentiment and theme analyses using the NetBase™ platform.

**Fig. 2:**
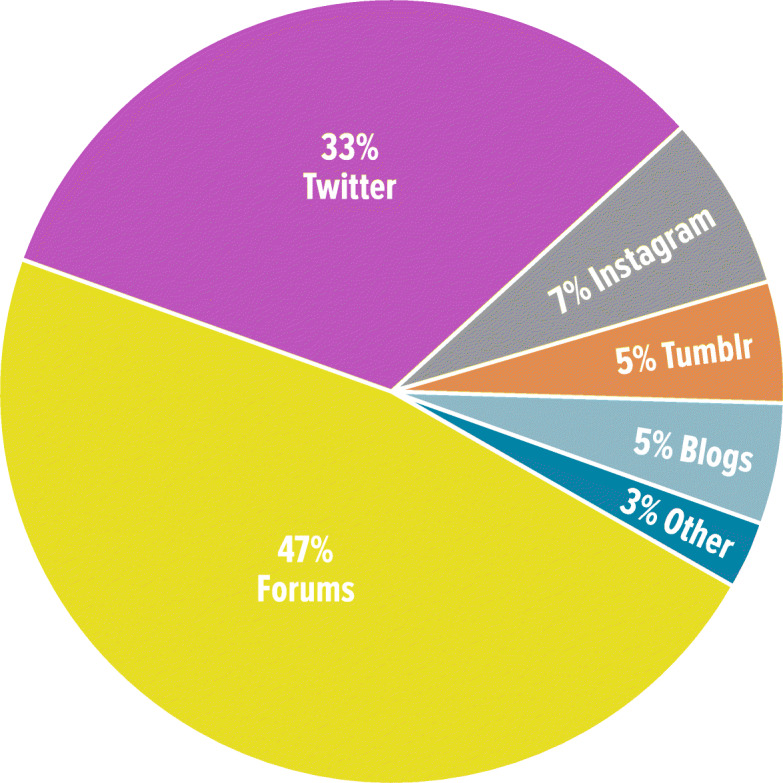
Sources of Social Media Posts (*N* = 107)

Based on information that was explicitly stated or inferred from content, 61.9% (39/63) of posts were written by females. Patients were responsible for 95.3% (102/107) of posts; the remaining 5 posts were from caregivers. All 107 posts included for quantitative analyses were from the United States. Based on available information, 93 posts were published from May 2017 to November 2019 (Supplementary Figure S1). Sources for the posts were primarily from online forums (47%) and Twitter (33%) (Fig. 2). A list of available websites for these sources is provided in Supplementary Table S2.

### Sentiment and theme analyses

**Fig. 3:**
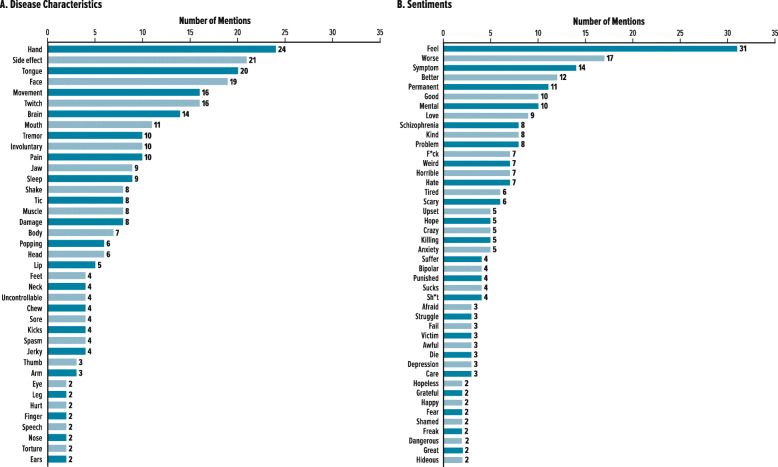
TD-Related Terms from Social Media Sources. Terms are presented in descending order of mentions; a single post might include > 1 term. **a**. Disease Characteristics. **b**. Sentiments

**Fig. 4:**
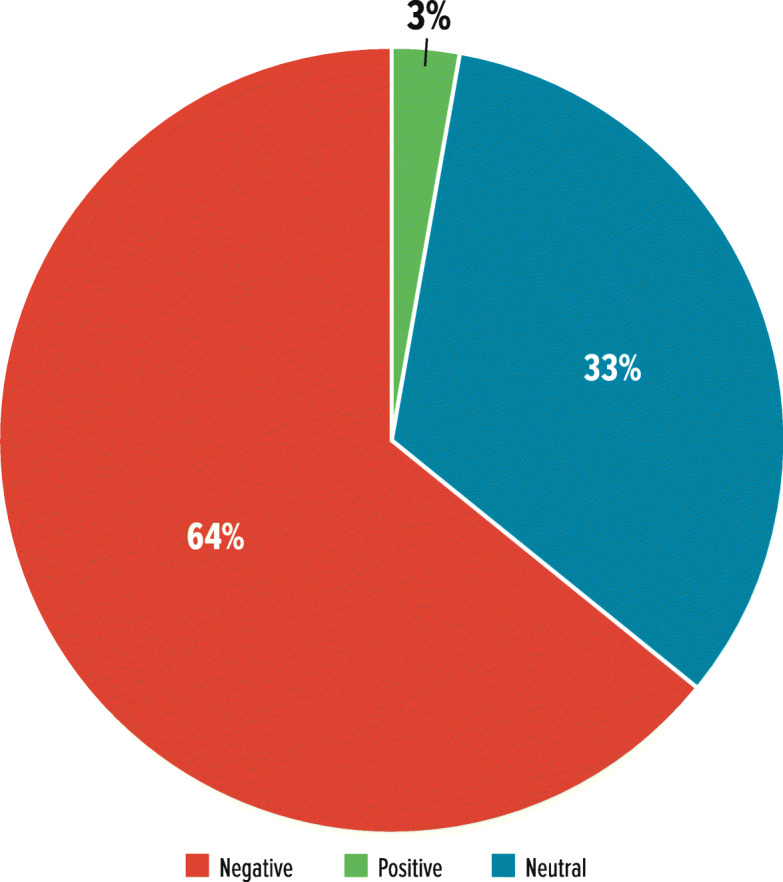
Sentiment Analysis

TD-related terms from social media sources included 84 terms that could be categorized as disease characteristics (*n* = 39) or sentiments (*n* = 45) (Fig. 3). Analysis of the most common sentiment-related terms (e.g. “feel” [*n* = 31], “worse” [*n* = 17], “symptom” [*n* = 14], “better” [*n* = 12]) indicated that 64% were negative, 33% were neutral, and 3% were positive (Fig. 4).

**Fig. 5:**
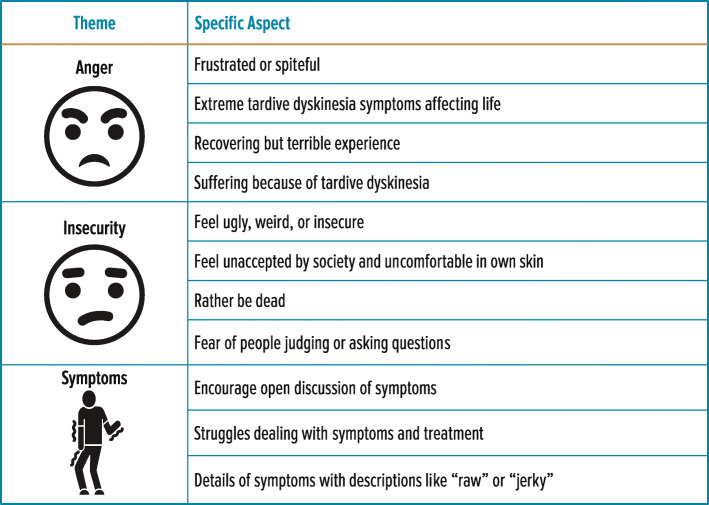
Theme Analysis

The posts were grouped into 3 major themes: anger, insecurity, and symptoms (Fig. 5). The theme of “anger” included posts from patients who were frustrated or spiteful about the condition. The main reason for anger was the negative impact of TD symptoms and movements (e.g., tics, chewing) on patients’ lives. Although some patients appeared to be recovering from their symptoms, they still expressed their experience in angry terms. Others indicated feelings of suffering due to TD. The theme of “insecure” included posts that discussed how TD movements made patients feel ugly, weird, or self-conscious. Patients expressed feeling unaccepted by society or uncomfortable in their own skin. Some feared being judged by others or being asked about their twitching. A few indicated that they would rather be dead than have TD. The theme of “symptoms” included posts in which patients described their symptoms (e.g., “raw” or “jerky”) and encouraged others to talk about their symptoms, as well as their struggles to find adequate treatment.

## Discussion

SML is an analytical method that can provide insight into how patients describe their TD symptoms and their feelings about having this disorder. PROs such as the PGIC have been used in clinical trials of approved TD medications, including valbenazine and deutetrabenazine [8–10, 16–18], and in RE-KINECT, a real-world study of possible TD in which patients were administered the EuroQoL 5-Dimension 5-Level questionnaire, the Sheehan Disability Scale, and a questionnaire about their involuntary movements and the impact of these movements on daily activities [4]. Because the emotions and sentiments of patients (and sometimes caregivers) on social media platforms are unsolicited and unfiltered, SML methods differ from these PROs and offer a more comprehensive context for understanding how patients experience TD and the unmet needs in this therapeutic space. As indicated in the recent FDA guidance on patient engagement for drug development, social media can allow access to difficult-to-reach populations, provide accurate and automatic capture of data, and possibly result in greater self-disclosure by patients [14].

SML is typically used in market research, but with a rigorous approach using health economics and outcomes research (HEOR) methodologies to assess the value proposition for an intervention, it can also guide patient-focused drug development and patient-centered treatment strategies [14, 15]. For example, an SML study of chronic obstructive pulmonary disease found that cough, mucus production, and shortness of breath were the symptoms of greatest concern for patients in terms of disease management [13]. In an SML study of dry eye, patients described the impact on work and other quality-of-life issues in addition to expressing frustration with suboptimal management of their symptoms [12]. By evaluating patient perspectives based on their own words, without any prompting from clinicians/investigators or limitations imposed by clinical trial protocols, these studies were able to gather fresh and unbiased patient perspectives that could complement findings based on more traditional research methods. For clinicians, familiarization with the terms and language used by patients on social media platforms may help guide discussions about symptoms, burden, and treatment options.

In this study, which is the first to apply SML methods to TD, almost all posts were from patients, with a few from caregivers. All posts originated in the United States, with approximately one-third from Twitter. Some assumptions about sex, race, and age group could be made based on photos or content of online posts, but no methods were available to verify demographic or socioeconomic data. However, given the various factors which contribute to the digital divide (e.g., age/generational status, gender, race, income, education) [19, 20], it should be noted that some key populations are probably not represented in this analysis (e.g., homeless patients, elderly patients, patients with intellectual or development disabilities). In addition, patients who are unaware of their TD are unlikely to be discussing their symptoms online and would therefore not be included in this type of study.

The results presented in this study, including how patients describe their TD symptoms and their feelings about those symptoms, are consistent with the types of data that the FDA has indicated as being appropriate for social media sources [14]. Given that TD can be a highly distressing and potentially disabling disorder [1, 21], it was perhaps surprising that 36% of online posts were assessed as neutral (33%) or positive (3%), although discerning the underlying reasons for neutrality or positivity was beyond the scope of this study. It is less surprising that many of the online posts analyzed in this study included negative terms and that 2 of the 3 major themes were “anger” and “insecurity”. Aspects within those themes included expressions of frustration, feelings of being unaccepted by society, and fear of being judged by others. Healthcare providers may help to offset some of these negative emotions by informing patients about the availability of approved TD treatments (e.g., valbenazine and deutetrabenazine) and by providing resources such as the “Talk About TD” website [22] and information for local support groups. Moreover, engaging with patients through social media may foster discussions about TD that can be both educational and supportive. Most importantly, simple follow-up questions about mental well-being during regular visits, coupled with referrals for psychosocial counseling as needed, would help clinicians gain better insights into the experiences of their patients and allow patients to feel that their concerns are being addressed.

### Limitations

This study had several limitations worth noting. First, a clinical diagnosis of TD in the users/patients could not be confirmed as TD was self-reported in online posts. Second, patients with TD are a heterogeneous population, but the posts in this report may not reflect the experience of all patients with TD. Additionally, the sample size was small, and as discussed earlier, certain patient populations may have been unable or unwilling to discuss their disease in online forums. Third, online posts often lack detail which may be the result of platform restrictions (e.g., character limits in Twitter). Finally, the perspective of caregivers was under-represented in this study and more research is needed to characterize the burden of TD on caregivers. More research is also needed to further understand the reasons why patients (and caregivers) choose to express their sentiments online and how healthcare providers can address these sentiments.

## Conclusions

This study offers an unfiltered glimpse into patients’ experiences with TD, which might have been more difficult to obtain in formal clinical or research settings. Although the study was limited by various methodological constraints, these patients’ experiences with TD—as described in social media posts expressing anger and insecurity—may help clinicians better understand the type of TD-specific care that patients may need.

## Supplementary Information

**Additional file 1: Figure S1.** Volume of Selected TD-Related Posts.

**Additional file 2: Table S1.** Criteria for Search Terms.

**Additional file 3: Table S2.** Websites Sources of TD-Related Posts Selected for Analysis.

## Data Availability

Although posts identified in this study were publicly available on social media platforms, the analysis excluded any personal identifiable information. Reasonable requests for data and material can be made by contacting the corresponding author.

## Abbreviatons

APA: American Psychiatric Association
FGA: first-generation antipsychotics
HEOR: health economics and outcomes research
PGIC: Patient Global Impression of Change
PROs: patient-reported outcomes
SGA: second-generation antipsychotics
SML: social media listening
TD: tardive dyskinesia
VMAT2: vesicular monoamine transporter 2

## References

[1] Jain R, Correll CU (2018). Tardive dyskinesia: recognition, patient assessment, and differential diagnosis. J Clin Psychiatry.

[2] Carbon M, Hsieh CH, Kane JM, Correll CU (2017). Tardive dyskinesia prevalence in the period of second-generation antipsychotic use: a meta-analysis. J Clin Psychiatry.

[3] Correll CU, Kane JM, Citrome LL (2017). Epidemiology, prevention, and assessment of tardive dyskinesia and advances in treatment. J Clin Psychiatry..

[4] Caroff SN, Yeomans K, Lenderking WR, Cutler AJ, Tanner CN (2020). RE-KINECT: a prospective study of the presence and healthcare burden of tardive dyskinesia in clinical practice settings. J Clin Psychopharmacol.

[5] Strassnig M, Rosenfeld A, Harvey PD (2018). Tardive dyskinesia: motor system impairments, cognition and everyday functioning. CNS Spectr.

[6] McEvoy JP (2019). Psychosocial implications of tardive dyskinesia in patients with mood disorders versus schizophrenia. J Clin Psychiatry.

[7] The American Psychiatric Association practice guideline for the treatment of patients with schizophrenia. Available at: https://www.psychiatry.org/psychiatrists/practice/clinical-practice-guidelines. Accessed 21 Dec 2020. 10.1176/appi.ajp.2020.177901 32867516

[8] O'Brien CF, Jimenez R, Hauser RA, Factor SA, Burke J, Mandri D, Castro-Gayol JC (2015). NBI-98854, a selective monoamine transport inhibitor for the treatment of tardive dyskinesia: a randomized, double-blind, placebo-controlled study. Mov Disord.

[9] Hauser RA, Factor SA, Marder SR, Knesevich MA, Ramirez PM, Jimenez R (2017). KINECT 3: a phase 3 randomized, double-blind, placebo-controlled trial of valbenazine for tardive dyskinesia. Am J Psychiatry.

[10] Fernandez HH, Factor SA, Hauser RA, Jimenez-Shahed J, Ondo WG, Jarskog LF (2017). Randomized controlled trial of deutetrabenazine for tardive dyskinesia: the ARM-TD study. Neurology..

[11] Anderson KE, Stamler D, Davis MD, Factor SA, Hauser RA (2017). Deutetrabenazine for treatment of involuntary movements in patients with tardive dyskinesia (AIM-TD): a double-blind, randomised, placebo-controlled, phase 3 trial. Lancet Psychiatry.

[12] Cook N, Mullins A, Gautam R, Medi S, Prince C, Tyagi N, Kommineni J (2019). Evaluating patient experiences in dry eye disease through social media listening research. Ophthalmol Ther.

[13] Cook NS, Kostikas K, Gruenberger JB, Shah B, Pathak P, Kaur VP (2019). Patients’ perspectives on COPD: findings from a social media listening study. ERJ Open Res.

[14] U.S. Department of Health and Human Services Food and Drug Administration. Patient-focused drug development: collecting comprehensive and representative input guidance for industry, Food and Drug Administration staff, and other stakeholders. Silver Springs: Center for Drug Evaluation and Research; 2020.

[15] Santos J, Palumbo F, Molsen-David E, Willke RJ, Binder L, Drummond M (2017). ISPOR code of ethics 2017 (4th edition). Value Health.

[16] Factor SA, Remington G, Comella CL, Correll CU, Burke J, Jimenez R (2017). The effects of valbenazine in participants with tardive dyskinesia: results of the 1-year KINECT 3 extension study. J Clin Psychiatry..

[17] Marder SR, Singer C, Lindenmayer JP, Tanner CM, Comella CL, Verghese C (2019). A phase 3, 1-year, open-label trial of valbenazine in adults with tardive dyskinesia. J Clin Psychopharmacol.

[18] Fernandez HH, Stamler D, Davis MD, Factor SA, Hauser RA, Jimenez-Shahed J (2019). Long-term safety and efficacy of deutetrabenazine for the treatment of tardive dyskinesia. J Neurol Neurosurg Psychiatry.

[19] Fang ML, Canham SL, Battersby L, Sixsmith J, Wada M, Sixsmith A (2019). Exploring privilege in the digital divide: implications for theory, policy, and practice. Gerontologist..

[20] Mitchell UA, Chebli PG, Ruggiero L, Muramatsu N (2019). The digital divide in health-related technology use: the significance of race/ethnicity. Gerontologist..

[21] Sreeram V, Shagufta S, Kagadkar F (2019). Role of vesicular monoamine transporter 2 inhibitors in tardive dyskinesia management. Cureus..

[22] Neurocrine Biosciences, Inc (2020). Talk About TD.

