# Supplementary figures and images for: Patient perspective of tardive dyskinesia: results from a social media listening study
Source: BMC Psychiatry. 2021 Feb 15;21:94. doi: 10.1186/s12888-021-03074-9 (PMC7885234; doi:10.1186/s12888-021-03074-9)

Number of Posts

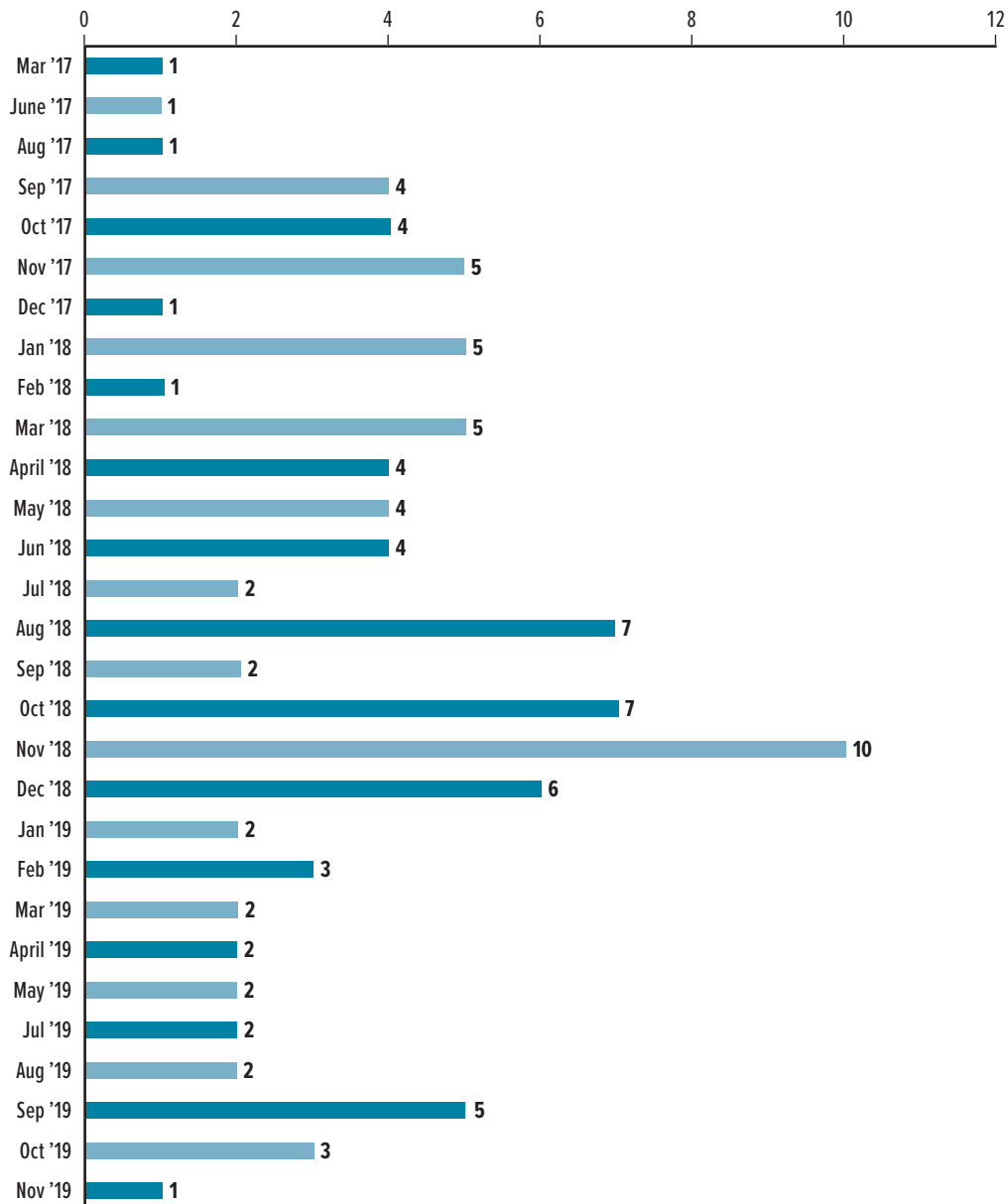

Supplement: Supplementary file 1 — Additional file 1: Figure S1. Volume of Selected TD-Related Posts. [file 12888_2021_3074_MOESM1_ESM.pdf]
