# Supplementary material for: Patient perspective of tardive dyskinesia: results from a social media listening study
Source: BMC Psychiatry. 2021 Feb 15;21:94. doi: 10.1186/s12888-021-03074-9 (PMC7885234; doi:10.1186/s12888-021-03074-9)
Supplement: Supplementary file 2 — Additional file 2: Table S1. Criteria for Search Terms. [file 12888_2021_3074_MOESM2_ESM.docx]

**Table S1. Criteria for Search Terms**

| **Primary Term(s):**  Tardive Dyskinesia, tardivedyskinesia, tardarive diskensia, tardarive dukanesia, tardarive dyskinesia, tardave disconesia, tardic dyskinesia, tardidive dyskinesia, tardif disconesia, tardiff dyskenisia, tardive syndrome, tardivesyndrome | **Excluded Term(s):**  Abilify, administration, ashawagandha, Austedo, bipartisan, Christine Blasey Ford, Christine Ford, Conyers, clozapine, dementia, democrat, democratic, democrats, deutetrabenazine, Donald, Donald Trump, dosage, edisylate, fumarate, haloperidol, hydrochloride, Ingrezza, Latuda, leftist, lineback, linebacker, Nancy, Nancy Pelosi, NBIX, Neurocrine, Neurocrine Biosciences, partisan, Pelosi, politics, political, precautions, prochlorperazine, quarterly, quetiapine, republican, Risperdal, Seroquel, SOM3355, term limits, tetrabenazine, Teva, The Donald, Thorazine, Tofranil, treatment market, Trump, valbenazine, Xenazine, ziprasidone, $NBIX, $Teva |
| --- | --- |
