# Supplementary material for: Patient perspective of tardive dyskinesia: results from a social media listening study
Source: BMC Psychiatry. 2021 Feb 15;21:94. doi: 10.1186/s12888-021-03074-9 (PMC7885234; doi:10.1186/s12888-021-03074-9)
Supplement: Supplementary file 3 — Additional file 3: Table S2. Websites Sources of TD-Related Posts Selected for Analysis. [file 12888_2021_3074_MOESM3_ESM.docx]

**Table S2. Websites Sources of TD-Related Posts Selected for Analysis**

| **Domain Name** |
| --- |
| [www.reddit.com](https://protect-us.mimecast.com/s/whF1CjRMLBHnlB7os7F0v9?domain=reddit.com) |
| psychcentralforums.com |
| computerbuildingforum.com |
| community.babycenter.com |
| forum.schizophrenia.com |
| [www.wordpress.com](https://protect-us.mimecast.com/s/7JsHClYLD0f2OVBRCY6UCA?domain=wordpress.com) |
| [www.tumblr.com](https://protect-us.mimecast.com/s/1zRBCn5X8lh7lPJ9i0FI24?domain=tumblr.com) |
| [www.instagram.com](https://protect-us.mimecast.com/s/i1TsCpYK7nfnQVj4svitPP?domain=instagram.com) |
| crazyboards.org |
| forums.studentdoctor.net |
| fourms.att.com |
| psychforums.com |
| [www.twitter.com](https://protect-us.mimecast.com/s/9nEJCrk5XpS8wk5Qs6JTjw?domain=twitter.com) |

TD, tardive dyskinesia.
